# Detecting methylation signatures in neurodegenerative disease by density-based clustering of applications with reducing noise

**DOI:** 10.1038/s41598-020-78463-3

**Published:** 2020-12-17

**Authors:** Saurav Mallik, Zhongming Zhao

**Affiliations:** 1 Center for Precision Health, School of Biomedical Informatics, The University of Texas Health Science Center at Houston, Houston, TX 77030 USA; 2 Human Genetics Center, School of Public Health, The University of Texas Health Science Center at Houston, Houston, TX 77030 USA; 3 Department of Psychiatry and Behavioral Sciences, McGovern Medical School, The University of Texas Health Science Center at Houston, Houston, TX 77030 USA

**Keywords:** Computational biology and bioinformatics, Molecular biology, Biomarkers

## Abstract

There have been numerous genetic and epigenetic datasets generated for the study of complex disease including neurodegenerative disease. However, analysis of such data often suffers from detecting the outliers of the samples, which subsequently affects the extraction of the true biological signals involved in the disease. To address this critical issue, we developed a novel framework for identifying methylation signatures using consecutive adaptation of a well-known outlier detection algorithm, density based clustering of applications with reducing noise (DBSCAN) followed by hierarchical clustering. We applied the framework to two representative neurodegenerative diseases, Alzheimer’s disease (AD) and Down syndrome (DS), using DNA methylation datasets from public sources (Gene Expression Omnibus, GEO accession ID: GSE74486). We first applied DBSCAN algorithm to eliminate outliers, and then used Limma statistical method to determine differentially methylated genes. Next, hierarchical clustering technique was applied to detect gene modules. Our analysis identified a methylation signature comprising 21 genes for AD and a methylation signature comprising 89 genes for DS, respectively. Our evaluation indicated that these two signatures could lead to high classification accuracy values (92% and 70%) for these two diseases. In summary, this framework will be useful to better detect outlier-free genetic and epigenetic signatures in various complex diseases and their developmental stages.

## Introduction

The past 2 decades have witnessed exponential growth of genetic and epigenetic data generation, which substantially helps the advancement of biological and biomedical research. However, analysis of these datasets often suffers from the exclusion of outliers, leading to a decrease of power for detecting the true genetic or epigenetic markers^1,2^. This issue has caused a great challenge on identifying the true outliers present in the complex data, and then remove them in the data process and analysis. In literature, this process of outlier removal has been typically overlooked. Accordingly, the data analysis might have led to inaccurate results or missing the true signals. In this study, we focused on development novel analytical strategy for outlier detection from DNA methylation data, and then applied to the real datasets for two representative neurodegenerative diseases.

Epigenetics refers to the study of genetic changes (e.g., gene expression) that do not involve the alternation at the DNA sequence level (e.g., DNA mutations), but they lead to the changes at the expression level or phenotype (e.g., disease or traits). The keyword “epigenetics” was first introduced in the early 1940s as a traditional (general) term by British embryologist Conrad Waddington to demonstrate the interactions between the genes and their products that promote the development and give rise to phenotype (observable qualities) of any organism. Since then, knowledge acquired from the epigenetics studies has transmuted in the domain of the genetics. So far, researchers have revealed various chemical alternations to DNA and then proteins denoted as histones which are connected to DNA very tightly in the nucleus through chromatin. These alternations can be detected while a specific gene is expressed either in the cell or the organism https://www.britannica.com/science/epigenetics^3^.

Epigenetics includes various factors such as histone modifications, DNA methylation^4–6^, microRNA^7^ and other types of RNA (e.g., 6mA, 4mC) regulation^8^, and protein expression modifications such as acetylation, sumoylation, ubiquitination, and phosphorylation^8–10^. DNA methylation regulation is a well-known epigenetic process. In mammalian genomes, 5-methylcytosine (5mC) methylation occurs when a methyl group is added to a cytosine, typically at the CpG dinucleotide^11,12^. These methylation and demethylation processes in cellular system are based on genetic factors, environmental factors, and their interactions^13,14^. Abnormal patterns of DNA methylation can lead to the creation and progression of various critical diseases^15–19^. The 5mC plays a key role in silencing X-chromosome and regulating gene expression at the specific locus or genome level^20,21^. Several DNA methyltransferase (denoted as DNMT) enzymes such as DNMT3A, DNMT3L, DNMT3B and DNMT1 catalyze the methylation^22,23^. The GC content and frequency of CpGs in a gene impact on the pattern of the methylation. For example, the CpG islands (denoted as CGIs), which are enriched CpG sites, are often hypo-methylated. The non-CGI sequences in the genome, which are the scattered CpGs in the genomic regions, are typically hyper-methylated^24^. Typically, approximately 80% of the CpGs in the genome are methylated, and de-methylation is a main mechanism to activate gene expression^25–28^. Hence, methylation leads to gene silencing^29–32^.

Another highly stable methyl cytosine variant next to 5mC is 5-hydroxymethylcytosine (5hmC)^27,33–35^. 5-Hydroxymethylcytosine (5hmC) is one of the most challenging topics in the field of epigenetics in the past 3–4 years. 5hmC has great potential for deep understanding of epigenetics in the brain tissue and its development. The oxidative product of 5mC is called 5hmC. The 5hmC mark was first reported in the T-Even bacteriophage about 7 decades ago^36^. So far, 5hmC changes have been found to be associated with several cancer and neurodevelopmental diseases such as Huntington’s disease and Alzheimer’s disease, suggesting it is a useful type of biomarkers for disease study^37^. Recently, 5hmC was detected in the brain of vertebrates as well as in other tissues^38–40^. In mice, 5hmC is reported to be abundant in the embryonic stem cells^41^. The abundance of 5hmC decreases upon differentiation^42,43^, but it increases again in the terminally differentiated cells (such as Purkinje neurons)^38^. The 5hmC is found in zygotes of rabbits, mice and bovines, and it is accumulated particularly in the paternal pro-nucleus along with a decrease of 5mC^44,45^. The various translocation (TET) protein-family members interfere with the level of 5hmC. An efficient chemical approach is currently being developed for measuring as well as labeling the 5hmC that represents the first map of distribution of 5hmC in a mouse brain, and its enrichment in the genes with a higher transcription^46^. The association of 5hmC with the specific gene-bodies at the time of differentiation and the maturation of neurons states that the 5hmC is spatially as well as temporally distributed in the brain tissue during the development of the brain. Transformation of 5mC into 5hmC is highly liable for the passive methylation. The 5hmC can be categorized into three sub-types according to their functions: 5hmC-A, 5hmC-B, and 5hmC-C. Among them, 5hmC-A primarily restrains the maintenance of DNA methylation during DNA replication^43^, 5hmC-B, which is prevented by the DNA repair protein, is used to active DNA demethylation^47–49^, 5hmC-C can obstruct the inclusion of the histone deacetylases, causing the development of the transcriptionally competent chromatin^50^.

So far, epigenetic marks such as DNA methylation and histone modifications have been extensively examined in the cellular system, including some large-scale epigenomic datasets such as The Roadmap Project^51^ and The Encyclopedia of DNA Elements (ENCODE) project^52,53^. These regulators play critical roles in the cause and the progression of diseases such as neurodegenerative diseases, including Alzheimer’s disease (AD) and Down syndrome (DS). The roles of 5mC and 5hmC in these diseases have also been documented^27,37,54,55^. There are some lines of evidence supporting the importance of 5mC and 5hmC in the pathogenesis of AD^56–59^ and DS^60^. However, in the previous studies, only 5mC labeled samples, not 5hmC labeled samples, were considered in data preparation. Interestingly, many biological data suffer from the outlier features, reducing the power to detect the true markers^1^. There is a need to remove those noisy features in the beginning of the data process and analysis. In this study, we applied a well-known density based noise removal clustering algorithm, DBSCAN (“density based clustering of applications with noise”), to remove noise from a 5mC and 5hmC methylation profile (GEO ID: GSE74486) in the tissue of frontal cortex (FC) neurons for AD and DS. This procedure is followed by differential methylation analysis using Limma method and gene module identification using hierarchical clustering. We here performed two comparative analyses for differential methylation: (1) AD vs the matched control samples considering its FC neuron samples, and (2) DS vs the matched control samples using its FC neuron samples. We then identified the gene signature for each comparative study. The module with the highest average correlation score was considered as a potential methylated signature for AD as well as DS. Furthermore, different cluster validity index measures, such as Dunn Index (DI), Scaled Connectivity (SC), Silhouette Width (SW), Cluster Coefficient (CC), Maximum Adjacency Ratio (MAR), Centralization (Ctz) and Heterogeneity (Hg), were estimated to determine the quality and efficiency of the clustering. To verify the resultant gene signature, we applied Random Forest (RF) classifier to generate the group classification performance of the underlying samples of the signature. In addition, KEGG pathway and Gene Ontology (GO) term analyses were carried out to assess the biological significance of the resultant signatures. The results from our DBSCAN analytical approach provide some important insights into the understanding of epigenetic regulation in AD and DS.

## Methods

**Figure 1:**
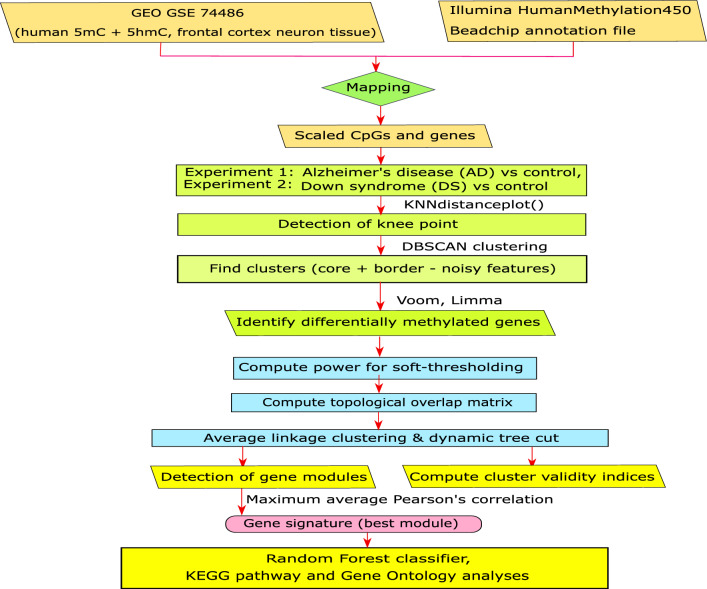
Flowchart of the DBSCAN framework and analysis.

In this study, we developed a new framework to identify the outlier-free DNA methylation signature for complex diseases. We then performed an extensive analysis for the classification of the methylation data for two neurodegenerative diseases, AD and DS, using the case and corresponding control samples (Fig. 1). The following steps were used to obtain the signature in our framework.

### Data collection and preliminary filtering

We used a CpG methylation profile (GEO ID GSE74486) in the tissue of human FC neurons^61^ (https://www.ncbi.nlm.nih.gov/geo/query/acc.cgi?acc=GSE74486, Accessed date: November 25, 2018). For the methylation analysis of AD, three AD FC neuron samples were used as the diseased samples, whereas eleven FC neuron samples were considered as the control samples. In case of the analysis of DS, nine DS FC neuron samples were used as the diseased samples, while the same eleven FC neuron samples were used as the control samples. In the GEO dataset GSE74486, the AD sample IDs were GSM1921521–GSM1921523 and the DS sample IDs were GSM1921524–GSM1921532, whereas normal sample IDs were GSM1921533–GSM1921543. The total number of Reference IDs (CpGs along with the IDs started with “rs” and with “ch”) was 485,577.

First, we eliminated the feature IDs starting with “rs” or “ch”, and kept only the CpGs. We then discarded the CpGs that had zero values in all samples or that had the missing value in any of the samples. Min-max normalization technique was used to normalize the data for each individual CpG. On the other hand, we collected the mapping information of CpG sites and official gene symbols (“UCSC_RefGene_Name”) through the annotation file “Illumina HumanMethylation450 BeadChip” (HumanMethylation450_15017482) (NCBI Ref. ID: GPL13534-11288). In the annotation file, there is either a one-to-many, many-to-one, or many-to-many relationship between CpGs and genes. We first chose the CpGs connected to each matched gene symbol, performing an average operation on all the CpG sites of each individual gene symbol to obtain a unique methylation data vector for each gene. Then, we conducted analyses for the two neurodegenerative diseases, AD and DS.

### Outlier detection through DBSCAN clustering algorithm

Since the sample size is small, applying the noise removal clustering algorithm prior to using any statistical test is extremely helpful. Specifically, we conducted DBSCAN clustering algorithm^62^ using those unique gene vectors and filtered out the noisy features for each analysis. In detail, for each disease, we preliminarily estimated the knee point through KNN distance plot, and that knee value was used as the eps-neighborhood value. Other parameters were set by default (e.g., MinPts = 5, weights = NULL, borderPoints = FALSE). This generated a few density-based clusters, while each contained core, border and outlier (noisy) features. We then discarded these noisy features and further proceeded with noise-free features for the statistical analysis. The resultant cluster plot obtained by the DBSCAN was provided to visualize those core, border and outlier features clearly.

In DBSCAN clustering, two required parameters were epsilon (*eps*) and minimum points (*MinPts*). DBSCAN is somewhat sensitive to parameter settings of *eps* and *MinPts*, but there is no specific theory that can completely guide the setting of its parameters^62–64^. The *eps*, the radius of the neighborhood around any point, was considered as $\epsilon$-neighborhood (epsilon-neighborhood) of the point. *MinPts* is the minimum number of neighbors inside the *eps* radius. If a point has a neighbor count value higher than or equal to *MinPts*, the point is stated as a core point. Whenever the number of the neighbors of a point is less than *MinPts*, but the point belongs to the $\epsilon$-neighborhood of a core point, the point is called as border point. On the other hand, if a point is neither a core point nor a border point, that point is considered as a noisy point. Our aim is to find the dense regions that can be evaluated by the number of objects (points) close to a specified point. In our study, we preliminarily estimated the *knee-point* through KNN (K-nearest neighbor) distance plot for each disease. To evaluate the *knee-point*, first KNN distances were computed and then sorted. Thereafter, they were scaled in between 0 and 1 ([0,1]). The derivative was then estimated. Finally, the first point in which the derivative was higher than a certain value, 1, was considered as * knee-point*. That corresponding scaled distance value of the * knee-point* was considered as *eps*-neighborhood value.

### Limma statistical analysis and identifying differentially methylated genes

After detection of the noise-free initial clusters through DBSCAN, we conducted Voom normalization^65^ and Limma statistical analysis^66–68^, consecutively on the features belonging to the noise-free clusters for identifying differentially methylated genes for the two experiments. In Limma, empirical Bayes and moderated t-statistic had been utilized for design. The moderated t statistic used in Limma could be demonstrated as follows:

1 $$\begin{aligned} {\tilde{t}}_{g} = \frac{1}{\sqrt{\frac{1}{n_{1}}+\frac{1}{n_{2}}}} \frac{{\hat{\beta }}_{g}}{{\tilde{s}}_{g}}, \end{aligned}$$

where *n* denotes the sample size ($n = n_{1} + n_{2}$); while ${\hat{\beta }}_{g}$ and ${\tilde{s}}_{g}^{2}$ refer to the contrast estimator and posterior sample variance for the feature *g*, respectively. The statistic for evaluating the contrast estimator for feature *g* can be denoted as follows:

2 $$\begin{aligned} {\hat{\beta }}_{g}|\sigma ^{2}_{g} \sim N(\beta _{g},\sigma ^{2}_{g}), \end{aligned}$$

where *N* is the normal distribution. However, the statistic to evaluate the posterior sample variance for the feature *g* is described as:

3 $$\begin{aligned} {\tilde{s}}_{g}^{2} = (d_{0}s_{0}^{2}+d_{g}s_{g}^{2})/(d_{0}+d_{g}), \end{aligned}$$

where $s_{0}^{2}$ and $d_{0}$ ($< \infty$) denote the prior variance and prior degrees of freedom, respectively, and $s_{g}^{2}$ and $d_{g}$ ($> 0$) are the experimental sample variance and experimental degrees of freedom for the feature *g*, respectively. After computing the t score by Limma, the p value for each feature (gene) *g* is evaluated. Whenever the p value of the gene is less than 0.05, the gene can be defined as differentially methylated gene.

### Gene module detection through hierarchical clustering

After obtaining the differentially methylated genes, we estimated the power for determining the soft-thresholding, and then applied this power to compute the adjacency matrix using Pearson’s correlation co-efficient. Next, we evaluated the topological overlap matrix (TOM) similarity and corresponding distance matrix from the adjacency matrix. Average linkage clustering and dynamic tree cut methods^69–73^ were applied consecutively to generate the gene modules highlighted by different colors. After obtaining the gene modules, we estimated the scores of several cluster validity index parameters such as Ball_hall, Davies_bouldin, Dunn, G_plus, Gdi11, Gdi12, Gdi31 and Ray_turi.

### Correlation analysis and detection of gene signature

After obtaining the gene modules, Pearson’s correlation coefficient (PCC) was computed among each gene-pair belonging to each gene module. Finally, the average correlation score for each cluster was obtained. The cluster that had the highest average PCC, was selected as the potential gene signature consisting of all differentially methylated genes.

### Evaluation of signature through sample group classification

To verify the classification performance of the resultant signature, we applied Random Forest (RF) classifier using k-fold cross-validation (CV) ($\hbox {k} = 2, 3, 4,\ldots$) on all the samples using all the features of the signature to classify two groups (AD/DS and control). The entire process was repeated many times. Finally, we computed the average classification accuracy and the area under the curve (AUC).

### Gene set enrichment analysis

In addition, we conducted gene set enrichment analysis using KEGG pathways and Gene Ontology (GO) terms available at DAVID online database (version 6.8)^74^. GO terms include three types, Biological Process (BP), Cellular Component (CC) and Molecular Function (MF). A KEGG pathway or GO term whose enrichment p value was less than 0.05, was considered as statistically significant. For more detail about the flowchart of the framework, see Supplementary Figure S5.

## Results

### Identification of non-redundant CpGs

We found a total of 485,577 features (IDs) in the initial analysis of the data (GEO GSE74486). After removing the redundant IDs that started with “rs” or “ch”, the number of CpGs was reduced to 482,421. We then eliminated the CpGs that had zero values in all samples or had missing value in any of the samples. After this filtering, we obtained a total of 435,662 CpGs. Next, we performed min-max normalization technique to scale all the data for each CpG. We collected the mapping information of CpG sites and official gene symbols through the annotation file (see “Methods”). We first selected those CpGs related to each matched gene symbol. We performed an average of the methylation values of all the CpG sites of each gene symbol, and obtained a unique methylation data vector for each gene. This resulted in a total of 20,247 gene vectors.

### Filtering noise-free features using DBSCAN clustering

**Figure 2:**
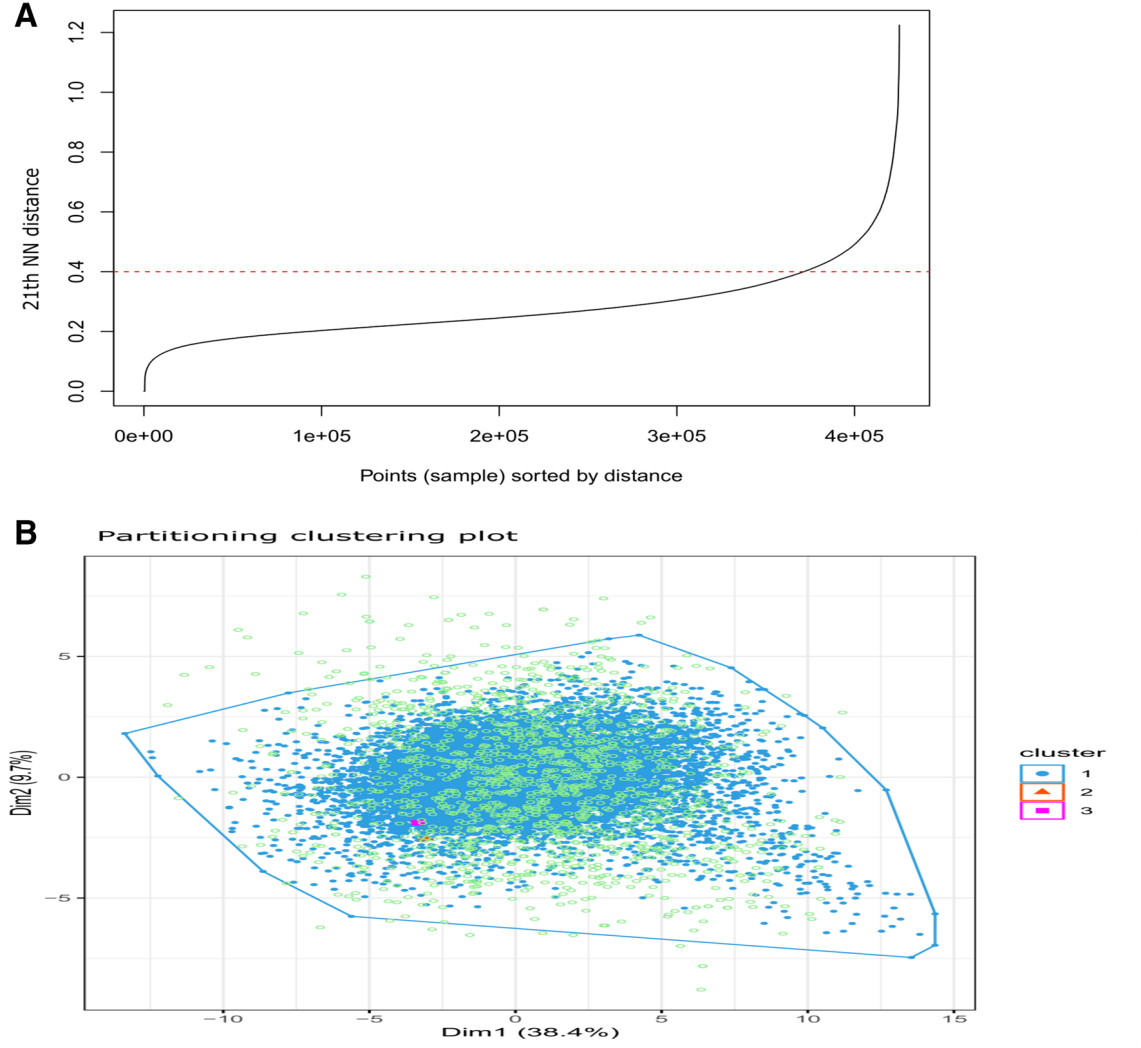
Combined figures of KNN distance plot and Partitioning clustering plot using DBSCAN clustering algorithm for DS vs control (FC neurons). (**A**) KNN distance plot to find knee point ($=0.4$ marked by red dotted line) used as EPS in DBSCAN clustering algorithms for DS vs control (FC neurons). (**B**) Partitioning clustering plot using DBSCAN clustering algorithm for DS vs control (FC neurons). Three clusters had been identified, among which the blue cluster contained 18,148 core (seed) features and 559 border features, the orange cluster had only 10 core features, and the violet cluster consisted of only 5 core features. In addition, a total of 1525 unclustered (outlier/noisy) features (denoted by light green dots) had been identified.

After applying the DBSCAN clustering algorithm on the 20,247 gene vectors, we filtered out the noisy features for the further analysis. In this regard, for the comparison of AD vs control samples, we first determined the knee point through KNN distance plot ($=0.4$, as marked by the red dotted line in Supplementary Figure S1). This knee point was used as eps-neighborhood value. The DBSCAN generated two clusters, one of which contained 19,592 core features and 206 border features. The second cluster had only 10 core features and no border feature, whereas the number of noisy features were 439 (Table 1). We then discarded these noisy features and further proceeded with the features ($=19{,}808$) belonging to these two clusters for the statistical analysis. The clusters with core and border features were denoted in blue and orange whereas outliers were depicted by green dots (Supplementary Figure S2). Similarly, in the comparison of DS vs control samples, we identified the knee point ($=0.4$ represented in Fig. 2A and Supplementary Figure S3) that was applied as eps-neighborhood value. This resulted in three clusters. One cluster consisted of 18,148 core and 559 border features while the remaining two clusters contained only 10 and 5 core features, respectively, with no border feature (Table 1). The number of noisy features were 1525 (Table 1). We then eliminated these noisy features, and further proceeded with the non-noisy features ($=18{,}722$) belonging to these three clusters for the statistical analysis. Figure 2B and Supplementary Figure S4 illustrated the three clusters containing the core, border and outlier features.

**Table 1:** Summary of DBSCAN clustering for outlier (noisy feature) removal prior to statistical test.

Comparison	Features	# outliers	# features		
			Cluster 1	Cluster 2	Cluster 3
$\hbox {AD}$ vs control	# border features	439	206	0	–
	# seed features	0	19,592	10	–
	Total	439	19,798	10	–
$\hbox {DS}$ vs control	# border features	1525	559	0	0
	# seed features	0	18,148	10	5
	Total	1525	18,707	10	5

*AD* Alzheimer’s disease, *DS* Down syndrome.

### Identification of differentially methylated genes using limma

**Figure 3:**
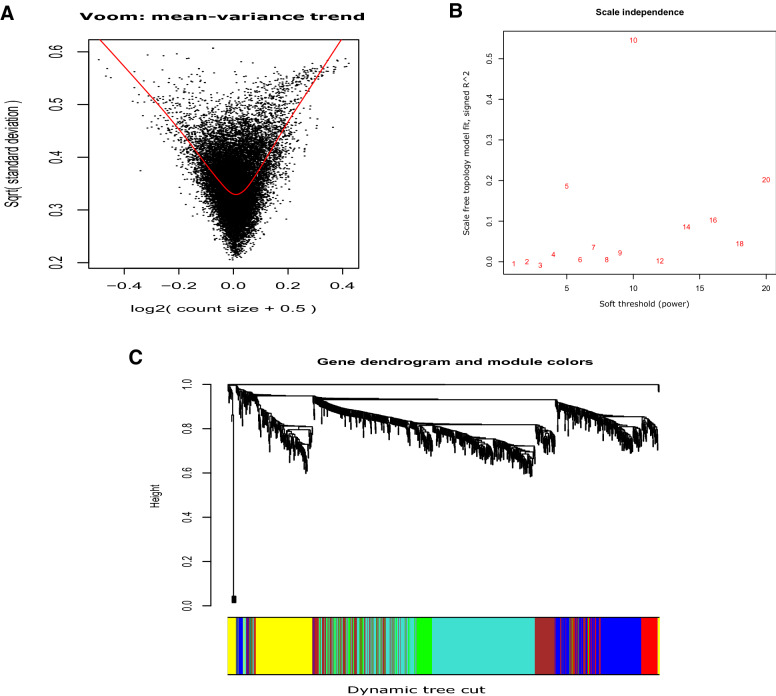
Plots for Voom normalization, power calculation for soft-thresholding and dendrogram for DS vs control. (**A**) Plot for Voom normalization for DS vs control. Voom normalization was used on the set of non-noisy features of the resultant clusters obtained from the pre-filtering analysis by DBSCAN clustering. (**B**) Power calculation for soft-thresholding in the comparison of DS vs control. This power computing is applied to ensure the scale free topology in the corresponding network. In this specific case, the final resultant power was set 10. (**C**) Dendrogram plot with color thresholding using dynamic tree cut method for the comparison of DS with control, while the x-axis denotes different gene modules represented by various colors and the y-axis shows the height of the tree (dendrogram).

After the pre-filtering analysis by DBSCAN clustering, we conducted Voom normalization and Limma statistical analysis, consecutively to identify differentially methylated genes for the two analyses. This resulted in a total of 229 differentially methylated genes, among which 133 were hyper-methylated and 96 were hypo-methylated in the comparison of the AD versus control samples. These numbers were 1062, among which 135 genes were hyper-methylated and the remaining 927 were hypo-methylated in the comparison of the DS versus control samples. Figure 3A presents the Voom plot for DS vs control.

### Detection of gene modules

After finding the set of the differentially methylated genes, we first estimated the power value of soft-thresholding, and then used the power to compute the adjacency matrix using Pearson’s correlation. Then the TOM score was computed and distance score was determined. Next, average linkage clustering and dynamic tree cut methods were used to identify gene modules. For the AD vs control analysis, we obtained a total of six modules. The number of participating differentially methylated genes for these six modules (illustrated by turquoise, brown, yellow, blue, red and green colors) were 83, 31, 28, 39, 21, and 27, respectively. Similarly, for the DS vs control analysis, using the power value (Fig. 3B), we generated a total of six modules. The number of participating differentially methylated genes for these six modules (colored by turquoise, yellow, brown, green, red and blue) were 380, 164, 172, 89, 71 and 184, respectively. Figure 3C shows the dendrogram plot for DS vs control. To determine the quality of the clustering in our proposed method, we evaluated several cluster validity indices such as Ball_hall, Davies_bouldin, Dunn, G_plus, Gdi11, Gdi12, Gdi31 and Ray_turi. For the AD vs control comparison, those values were 0.331, 5.205, 0.082, 0.136, 0.082, 0.363, 0.394 and 14.018, respectively (Table 2), whereas for the DS vs control comparison, those scores were 0.221, 3.040, 0.071, 0.089, 0.071, 0.325, 0.257 and 5.746, respectively (Table 3).

**Table 2:** Comparison of various cluster validity indices between our proposed method and k-means clustering for AD vs control.

Cluster validity index	Proposed method	K-means clustering
Ball_hall	0.331	0.111
Davies_bouldin	5.205	2.090
Dunn	0.082	0.009
G_plus	0.136	0.070
Gdi11	0.082	0.009
Gdi12	0.363	0.068
Gdi31	0.394	0.209
Ray_turi	14.018	2.229

Higher value signifies better than the other value in the same row (cluster validity index).

**Table 3:** Comparison of various cluster validity indices between our proposed method and k-means clustering for DS vs control.

Cluster validity index	Proposed method	K-means clustering
Ball_hall	0.221	0.192
Davies_bouldin	3.040	2.259
Dunn	0.071	0.014
G_plus	0.089	0.084
Gdi11	0.071	0.014
Gdi12	0.325	0.085
Gdi31	0.257	0.233
Ray_turi	5.746	2.770

Higher value signifies better than the other value in the same row (cluster validity index).

### Correlation analysis and methylation signature detection

**Figure 4:**
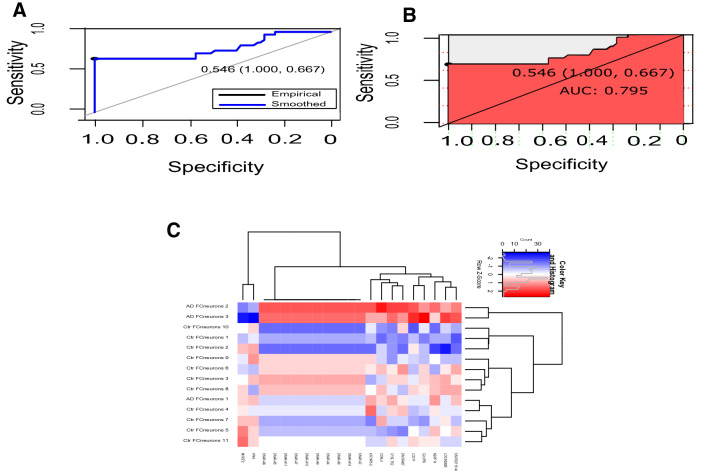
Area under the curve (AUC) result with 2-fold cross-validation and heatmap of gene signature for AD vs control. (**A**) Empirical and smoothed patterns for specificity vs sensitivity plot in AUC. (**B**) AUC plot (AUC value $=0.795$). (**C**) Heatmap for the cluster 5 (gene signature represented in red color) containing 19 hyper-methylated and 2 hypo-methylated genes for AD vs control, where “AD” and “ctr” on the x-axis stand for Alzheimer’s disease samples and control samples, respectively.

First, PCC was calculated between the pairwise genes in each cluster. The average PCC values of these six clusters for AD vs control, were 0.054, 0.446, 0.228, 0.154, 0.524 and 0.122, respectively. The fifth (red colored) cluster, which had the highest average PCC ($=0.524$), was chosen as the “gene-signature”. The gene-signature consisted of a total of 21 differentially methylated genes. Among them, 19 genes were hyper-methylated genes (*CDKL4, C2orf78, SNORD115-44, CYSLTR2, SNORA67, KRTAP5-2, LCE1F, LOC642826, SNAR-A14, SNAR-A3, SNAR-A6, SNAR-A4, SNAR-A9, SNAR-A10, SNAR-A7, SNAR-A11, SNAR-A5, SNAR-A8* and *NBPF14*), whereas the remaining 2 genes were hypo-methylated genes (*MIR572* and *IFNK*). The heatmap for the corresponding cluster is represented in Fig. 4C. Similarly, PCC values for DS vs control were measured between the pairwise genes in each cluster. The average correlation scores of these six clusters were 0.747, 0.327, 0.689, 0.747, 0.746 and 0.724, respectively. We selected the fourth module as the potential “gene signature” because of the lower number of participating genes ($=89$) in the fourth module, even though both the first (turquoise) and fourth (green) modules had the highest average correlation ($=0.747$). This DS signature included 89 differentially methylated genes, all of which were hypo-methylated genes. For details about the 89 genes, see Supplementary Table S1.

### Classification analysis of methylation signature

To verify the classification performance of the resultant signature, we applied Random Forest classifier through k-fold cross-validations (CVs) on all samples using all of its participating features to classify two groups (AD or DS, and control). The entire process was repeated 10 times. For the experiment: AD vs control, we computed accuracy and other evaluation metrics with three types of cross validations (CVs): 2-fold, 4-fold and 5-fold CV. The average accuracy values for these three types of CVs were 92.10%, 92.90%, and 92.90%, respectively, while the AUCs for these three types of CVs were 0.795, 0.783, and 0.771, respectively (Fig. 4A,B, Supplementary Figures S6, S7). For the comparison of the DS vs control samples, we used 2-fold, 5-fold and 8-fold CVs. For the 2-fold CV, we obtained 70.00% average accuracy and 0.664 AUC, whereas for 5-fold CV, these were 70.50% and 0.676, respectively and for the 8-fold CV, these were 70.00% and 0.673, respectively. The precision scores for these three CVs were 73.60%, 78.30% and 75.40%, respectively. Table 4 summarizes the classification accuracy, AUC and precision values for each disease analysis.

**Table 4:** Classification accuracy, area under the curve (AUC), and precision by cross-validation (CV) in two comparisons.

Comparison	$\hbox {CV}$ fold	Avg accuracy	$\hbox {AUC}$	Avg. precision
$\hbox {AD}$ vs control	2 fold	0.921	0.795	0.967
	4 fold	0.929	0.783	1
	5 fold	0.929	0.771	1
$\hbox {DS}$ vs control	2 fold	0.700	0.664	0.736
	5 fold	0.705	0.676	0.783
	8 fold	0.700	0.673	0.754

*CV* cross-validation, *AUC* area under the curve, *AD* Alzheimer’s disease, *DS* Down syndrome.

### Gene set enrichment analysis

In the gene set enrichment analysis, we obtained many statistically significant KEGG pathway and GO terms. Many participating genes belonging to the gene signature had fallen into those pathways and GO terms. Among those significantly enriched pathways and GO terms, many are related to the underlying biology of AD and DS. For example, *CYSLTR2* and *LCE1F* involved with a GO-CC term, GO:0016021 Integral component of membrane (p value 0.031), whose association with AD was reported in Smith et al.^75^. Thus, *CYSLTR2* and *LCE1F* are indirectly connected with AD through that GO term. On the other hand, *CALML6, OR8G1* and *OR52H1* were associated with a KEGG pathway, hsa04740: Olfactory transduction (p value $5.51 \times 10^{-5}$), whose interaction in DS was recently reported in an article by Cecchini et al.^76^. Hence, these three genes were indirectly linked to DS through the pathway.

Other important significant pathways and GO terms were mentioned next. In the case of AD vs control comparison, a gene *LCE1F* was associated with five GO terms, including two GO biological pathways [GO:0031424 Keratinization (p value 0.031) and GO:0018149 Peptide cross-linking (p value 0.033)], another two were GO cellular component terms [GO:0016021 Integral component of membrane (p value 0.031) and GO:0001533 Cornified envelope (p value 0.027)], and one GO molecular function term [GO:0005198 Structural molecule activity (p value 0.011)]. Table 5 summarizes the resultant significant GO terms with enrichment p value for AD versus control analysis. For DS vs control, *ADH1B* was involved in two KEGG pathways, hsa05204: Chemical carcinogenesis (p value 0.003) and hsa00982: Drug metabolism-cytochrome P450 (p value 0.011). *HAMP, PGLYRP1, TINAG* and *TINAGL1* were associated in a GO-BP term, GO:0006955 Immune response (p value $3.25\times 10^{-10}$). *CX3CR1* was connected with two KEGG pathways, hsa04060: Cytokine–cytokine receptor interaction (p value $7.49\times 10^{-4}$) and hsa04062: Chemokine signaling pathway (p value 0.044), and two GO-BPs, GO:0006935 Chemotaxis (p value $8.73\times 10^{-6}$) and GO:0070098 Chemokine-mediated signaling pathway (p value $2.60\times 10^{-5}$). *TNFRSF17* gene had fallen into the KEGG pathway, hsa04060: Cytokine–cytokine receptor interaction (p value $7.49\times 10^{-4}$) and a GO-MF, GO:0004872 Receptor activity (p value $2.21\times 10^{-5}$). *PGLYRP1* was connected with a GO-CC term, GO:0005576 Extracellular region (p value $2.40\times 10^{-9}$). Table 6 summarizes the resultant significant GO terms with enrichment p value for DS versus control analysis.

**Table 5:** Top significant GO terms enriched with the Alzheimer’s disease specific genes.

$\hbox {Gsig}^{\text{a}}$	GO $\hbox {term}^{\text{b}}$	Enrichment p value
*CYSLTR2*	GO-CC: GO:0016021 Integral component of membrane	0.031
*LCE1F*	GO-MF: GO:0005198 Structural molecule activity	0.011
	GO-CC: GO:0001533 Cornified envelope	0.027
	GO-BP: GO:0031424 Keratinization	0.031
	GO-BP: GO:0016021 Integral component of membrane	0.031
	GO-BP: GO:0018149 Peptide cross-linking	0.033

$^{\text{a}}$Gsig: genes belonging to the gene signature. $^{\text{b}}$For GO terms, it has three domains: Biological Process (BP), Cellular Component (CC), and Molecular Function (MF).

**Table 6:** Top significant KEGG pathways and GO terms enriched with Down syndrome specific genes.

$\hbox {Gsig}^{\text{a}}$	KEGG pathway or GO $\hbox {term}^{\text{b}}$	Enrichment p value
*ADH1B*	KEGG pathway: hsa05204: Chemical carcinogenesis	$3.24\times 10^{-3}$
	KEGG pathway: hsa00982: Drug metabolism-cytochrome P450	0.011
*CALML6*	KEGG pathway: hsa04740: Olfactory transduction	$5.51\times 10^{-5}$
*CD33*	GO-MF: GO:0004872 Receptor activity	$2.21\times 10^{-5}$
*CLEC2B*	GO-MF: GO:0030246 Carbohydrate binding	$5.26\times 10^{-9}$
*CX3CR1*	GO-BP: GO:0006935 Chemotaxis	$8.73\times 10^{-6}$
	GO-BP: GO:0070098 Chemokine-mediated signaling pathway	$2.60\times 10^{-5}$
	KEGG pathway: hsa04060: Cytokine–cytokine receptor interaction	$7.49\times 10^{-4}$
	KEGG pathway: hsa04062: Chemokine signaling pathway	0.044
*HAMP*	GO-BP: GO:0006955 Immune response	$3.25\times 10^{-10}$
*LCE3A*	GO-BP: GO:0030216 Keratinocyte differentiation	$9.70\times 10^{-4}$
*OR8G1*	KEGG pathway: hsa04740: Olfactory transduction	$5.51\times 10^{-5}$
*OR52H1*	KEGG pathway: hsa04740: Olfactory transduction	$5.51\times 10^{-5}$
*PGLYRP1*	GO-BP: GO:0006955 Immune response	$3.25\times 10^{-10}$
	GO-CC: GO:0005576 Extracellular region	$2.40\times 10^{-9}$
*RARRES1*	GO-CC: GO:0016021 Integral component of membrane	$1.59\times 10^{-11}$
*TAS2R41*	GO-BP: GO:0001580 Detection of chemical stimulus involved in sensory perception of bitter taste	$2.52\times 10^{-5}$
*TINAG*	GO-BP: GO:0006955 Immune response	$3.25\times 10^{-10}$
*TINAGL1*	GO-BP: GO:0006955 Immune response	$3.25\times 10^{-10}$
*TNFRSF17*	GO-MF: GO:0004872 Receptor activity	$2.21\times 10^{-5}$
	KEGG pathway: hsa04060: Cytokine–cytokine receptor interaction	$7.49\times 10^{-4}$

$^{\text{a}}$Gsig: genes belonging to the gene signature. $^{\text{b}}$For GO terms, it has three domains: Biological Process (BP), Cellular Component (CC), and Molecular Function (MF).

### Comparison with other method

In addition, we provided a comparative study of the scores of the eight cluster validity index measures between our proposed method and a well-known existing clustering method, k-means^77^. Of note, in the case of k-means clustering, we provided same cluster size that was estimated in our proposed method. For the experiment of AD vs control, Ball_hall scores for our proposed method and k-means clustering method were 0.331 and 0.111, respectively, whereas Davies_bouldin scores for them were 5.205 and 2.090, respectively. The detailed information for these eight measures for our proposed method and k-means for AD vs control and DS vs control was provided in Tables 2 and 3, respectively. In summary, we obtained better scores in our proposed DBSCAN based method than k-means for all of these eight validity index measures, indicating that our method has better performance than k-means clustering.

### Validation

For independent validation of our findings obtained in the analysis of DS vs control in FC neuron tissue, we used another 5mC and 5hmC methylation profile of the same disease (DS). This disease is from the same GEO accession ID (GSE74486) but with different tissue (cerebellum). To validate our resultant gene signature obtained by previous dataset, first we selected all the features belonging to the gene signature ($=89$), and then identified the corresponding sub-data of those features from the external dataset. Next, we applied several types of CVs (2-fold and 8-fold) on all the samples and then applied Random Forest classifier to classify two classes (diseased and normal groups) with the repetition of 10 times. For the 2-fold CV, we obtained 97.00% average accuracy, while for the 8-fold CV, the average accuracy was 97.80%. This validation analysis supported that our resultant gene signature provided excellent average classification accuracy in the similar methylation data.

Furthermore, we applied our entire proposed framework on the second new dataset (DS vs control: cerebellum tissue), and evaluated the average accuracy and AUC. Specifically, there were initially 482,421 features (IDs) in the beginning of the analysis along with 13 DS cerebellum and ten control samples. We initially selected 443,020 CpGs, and then obtained 20,252 gene-vectors from them. Next, in DBSCAN, four resultant clusters were generated of which one cluster consisted of 16,736 core and 753 border features, while the number of core features for the remaining three clusters were 5, 10 and 5, respectively, Notably, we found no border feature for these three remaining clusters. The number of outlier features were 2743. We then selected only the non-outlier features belonging to these four clusters ($=17{,}509$) for the next analysis. Moreover, we identified 1467 differentially methylated genes of which 607 were hyper-methylated and remaining 860 were hypo-methylated. A total of two gene modules was then detected by dynamic tree cut method of which 701 genes included in blue module and 762 genes in turquoise module (Supplementary Figure S11). Turquoise cluster, which had higher average PCC value ($=0.742$) than the other cluster was denoted as potential gene signature. This signature had 762 genes. We applied 8-fold CV on all the samples of those 762 features and then used Random Forest classifier with a repetition of 10 times. We obtained 82.60% average accuracy, 86.20% average sensitivity, 78.00% average specificity, 83.60% average precision, and 0.818 AUC value (Supplementary Figure S8).

### Generalized cis-regulatory enrichment analysis

We performed the generalized cis-regulatory enrichment analysis (i-cisTarget) using its web tool (https://gbiomed.kuleuven.be/apps/lcb/i-cisTarget/)^78,79^ on the resultant 89-gene signature for DS vs control (FC Neuron). Specifically, we used the 89 genes as the input to the i-cisTarget tool. We found 16 enriched features containing the normalized enrichment score (NES) $> 3.0$. Among these 16 enriched features, top three measured by NES score were 1) “GSM1208590_batch1_chrom1_LoVo_ARNT_PassedQC_peaks_hg19” (NES 4.36), 2) “GSM1208674_batch1_chrom1_LoVo_SMAD2_PassedQC_peaks_hg19” (NES 3.92), and 3) “GSM1208673_batch1_chrom1_LoVo_RXRA_PassedQC_peaks_hg19” (NES 3.82). The barplot of p value vs AUC in the TF binding sites for the prediction of regulatory features and cis-regulatory modules for the 89-gene signature was represented in Supplementary Figure S9(A), while the plot of #predicted regions vs rank in the best (topmost) feature was also illustrated in Supplementary Figure S9(B). The significantly high ranked regions (mentioned in Supplementary Figure S9) in UCSC Genome Browser for the prediction of regulatory features and cis-regulatory modules for the 89-gene signature was also shown in Supplementary Figure S10. The seventeen significantly highly ranked regions for the topmost feature were provided in Supplementary Table S2. For example, top three region IDs were chr3-reg108831, chr3-reg108833 and chr3-reg108832, while their associated gene was *RARRES1*, as part of the 89-gene signature. Next four region IDs were chr12-reg6728, chr12-reg6726, chr12-reg6725 and chr12-reg6724 whose associated gene was *LTBR* that was part of the 89-gene signature. Notably, the list of 16 enriched features obtained from the generalized cis-regulatory enrichment analysis for the 89-gene signature by i-cisTarget web tool was mentioned in Supplementary File S1.

### Additional analysis

**Figure 5:**
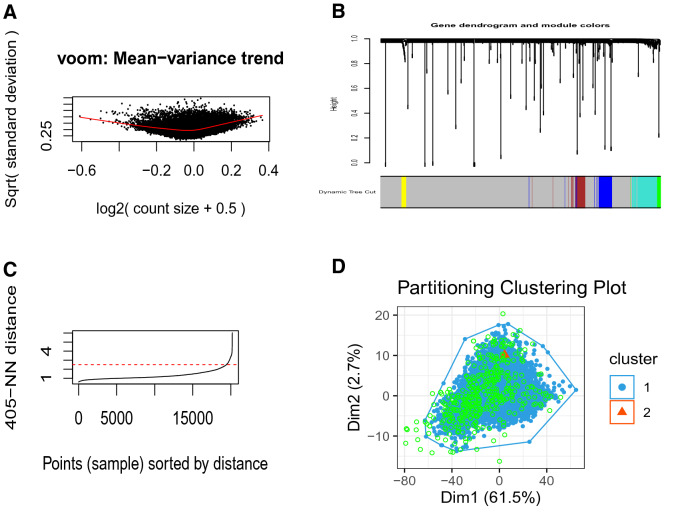
Plots for Voom normalization, dendrogram, KNN distance and Partitioning clustering using DBSCAN clustering algorithm for the dataset having NCBI GEO ID: GSE134379 (AD vs control in CBL). (**A**) Plot for Voom normalization for additional data (AD vs control in CBL). Voom normalization was used on the set of non-noisy features of the resultant clusters obtained from the pre-filtering analysis by DBSCAN clustering. (**B**) Dendrogram plot with color thresholding using dynamic tree cut method for the comparison of AD with control in CBL. (**C**) KNN distance plot to find knee point ($=2.5$ marked by red dotted line) used as EPS in DBSCAN clustering algorithms for AD vs control in CBL. (**D**) Partitioning clustering plot using DBSCAN clustering algorithm for AD vs control in CBL. Two clusters had been identified, among which the blue cluster contained 19,711 core (seed) features and 71 border features, and the red cluster had only 10 core features. In addition, a total of 398 unclustered (outlier/noisy) features (denoted by light green dots) had been identified.

The sample size in the previous validation is small, but the methylation data were generated from Fc neuron directly. To further validate our method, we used another recent real-life Alzheimer’s Disease (AD) data with larger sample size [NCBI GEO ID: GSE134379]. This data consisted of 4,11,157 CpG cites (features) and a total of 404 samples including 225 AD samples and 179 control samples from cerebellum (CBL) brain region of Illumina 450K methylation array. We applied our proposed method on this dataset. After the removal of redundant CpGs and the mapping between CpGs and corresponding genes, we obtained a total of 20,190 gene vectors (non-redundant features). After applying the DBSCAN clustering algorithm on the 20,190 gene vectors, we discarded the noisy features. In this regard, we first estimated the knee point through KNN distance plot ($=2.5$, as marked by the red dotted line in Fig. 5C). This knee point was used as eps-neighborhood value. The DBSCAN generated two clusters, one of which consisted of 19,711 core features and 71 border features. The second cluster contained only 10 core features while no border feature. Finally, the number of noisy features were 398. We then omitted these 398 noisy features and further proceeded with the remaining features ($=19{,}792$) belonging to these two clusters for the statistical analysis. Figure 5D showed the two clusters containing the core, border and outlier features. Thereafter, we determined a total of differentially methylated genes using Limma–Voom statistical analysis. This resulted in a total of 887 differentially methylated genes (Fig. 5A). Thereafter, by using the soft-thresholding, adjacency matrix using Pearson’s correlation, TOM score, distance score, average linkage clustering and dynamic tree cut methods, respectively, on these 887 differentially methylated genes, we detected five gene modules. The number of participating differentially methylated genes for these modules (illustrated by blue, brown, green, turquoise and yellow colors in Fig. 5B) were 50, 37, 10, 77 and 15, respectively, while other genes are unclustered. As part of quality measurement of the clustering in our proposed method, the values of eight cluster validity indices namely Ball_hall, Davies_bouldin, Dunn, G_plus, Gdi11, Gdi12, Gdi31 and Ray_turi were 1.767, 3.461, 0.069, 0.245, 0.069, 0.471, 0.207 and 14.887, respectively. Notably, the yellow cluster which had the highest average PCC ($=0.749$), was chosen as the gene-signature. The gene-signature consisted of a total of 15 Differentially methylated genes. The average accuracy values for AD vs control classes for the evolved gene signature for these 2-fold, 4-fold and 5-fold CVs were 76.94% ($\pm 2.76\%$), 79.49% ($\pm 1.75\%$), and 80.87% ($\pm 2.10\%$), respectively, while the AUCs for these three types of CVs were 0.817, 0.819, and 0.834, respectively.

## Discussion

So far, methylation gene signatures have been reported in many diseases or cellular conditions. They include two highly stable methylation variants, 5mC and 5hmC, that play some roles in several neurodegenerative diseases, such as AD and DS. Specially, one primary concern regarding the large but complex biological data analysis is the reduction of the noise. To meet this strong challenge, in this study, we proposed a new framework to identify outlier-free DNA methylation signatures through the utilization of the well-reputed DBSCAN clustering algorithm and hierarchical clustering, and applied it for the 5mC and 5hmC labeled methylation data (GEO ID: GSE74486) in the tissue of FC neuron for two neurodegenerative diseases: AD and DS. We first performed various pre-filtering analyses to initially remove the redundant CpGs. In this study, two sets of analyses had been conducted, (1) AD vs control, and (2) DS vs control, both using the methylation data from the FC neuron tissue, which is a critical tissue to study AD and DS. In our study, we preliminarily estimated the knee-point through KNN distance plot for each disease^62^. Next, the evolved knee-point would be used as a parameter *eps* in the next step, DBSCAN clustering algorithm where default settings of other parameters were also set to discard the outliers from the methylation data. Since the sample size was not very high, the DBSCAN was highly effective to filter out the outliers. Interestingly, DBSCAN is somewhat sensitive to parameter settings specially in *eps* and *MinPts*, but there is no specific theory which can provide entire guidance regarding the setting of its parameters^62–64^. However, Limma statistical method was then used to identify differentially methylated genes. Consecutive utilization of average linkage clustering and dynamic tree cut generated some colored gene modules ($=6$ for both the experiments). The module cluster having best average correlation score was considered as a potential methylated gene signature for AD (21-gene signature) as well as DS (89-gene signature). We obtained satisfactory classification accuracy for disease group from these signatures (92% for AD vs control, and 70% for DS vs control). Our proposed method had better performance than k-means clustering in terms of various cluster validity index measures.

There are a couple of reasons of obtaining big difference in the results: (1) the imbalanced dataset (i.e., higher number of features and very low number of samples) and (2) noise found in the datasets. The total number of initial features was 4,85,577, while total number of samples used for AD vs control experiment was 14 and total number of samples used for DS vs control experiment was 20. Thus, due to these extreme imbalanced datasets, the intermediate result up to the signature detection might be varied. For example, use of statistical test with smaller population size of samples are difficult. As a result, classification accuracy was be varied highly. The second reason is high noise in the dataset. To reduce noisy features, we applied DBSCAN clustering technique initially to detect noisy features for improve the later results. For the additional dataset (AD vs control in CBL brain region), we also obtained good average accuracy ($\sim 80\%$) to detect the two class classification for the respective signature genes that is obviously a good indication of the validation of the proposed method.

However, as future work, We will need to integrate the other types of data (e.g., gene expression, copy number variation, chromatin remodeling, etc.) in our analysis. Finally, our resultant outlier-free signatures might be useful for the potential detection of onset or progression of neurodegenerative diseases, as methylation is critical in such diseases.

## Supplementary information

Supplementary Information 1

Supplementary Information 2

Supplementary Information 3

Supplementary Information 4
