# Supplementary material for: Detecting methylation signatures in neurodegenerative disease by density-based clustering of applications with reducing noise
Source: Sci Rep. 2020 Dec 17;10:22164. doi: 10.1038/s41598-020-78463-3 (PMC7747741; doi:10.1038/s41598-020-78463-3)
Supplement: Supplementary file 1 — Supplementary Information 1 [file 41598_2020_78463_MOESM1_ESM.docx]

We performed the generalized cis-regulatory enrichment analysis (i-cisTarget) using its web tool (https://gbiomed.kuleuven.be/apps/lcb/i-cisTarget/) on the resultant 89-gene signature for DS vs control (FC Neuron). To do this, we used the 89 genes as input to the i-cisTarget tool. We found 16 enriched features the normalized enrichment score (NES) > 3.0 and other default parameters.

Those 16 features were

1. `` GSM1208590_batch1_chrom1_LoVo_ARNT_PassedQC_peaks_hg19" (NES 4.36),
2. ` GSM1208674_batch1_chrom1_LoVo_SMAD2_PassedQC_peaks_hg19" (NES 3.92),
3. `` GSM1208673_batch1_chrom1_LoVo_RXRA_PassedQC_peaks_hg19" (NES 3.82),
4. ``ENCFF001TXR" (NES 3.73),
5. `` GSM1208642_batch1_chrom1_LoVo_KLF5_PassedQC_peaks_hg19" (NES 3.71),
6. `` GSM1208644_batch1_chrom1_LoVo_LHX2_PassedQC_peaks_hg19" (NES 3.39),
7. ``ENCFF001TXC" (NES 3.37),
8. ``ENCFF001VLU" (NES 3.31),
9. ``ENCFF001TVA" (NES 3.28),
10. ``ENCFF001TWB" (NES 3.26),
11. ``ENCFF001TXQ" (NES 3.26),
12. ``ENCFF001UNV" (NES 3.15),
13. `` GSM1208742_batch2_chrom1_LoVo_FEV_PassedQC_peaks_hg19" (NES 3.08),
14. ``ENCFF001TYF" (NES 3.07),
15. ``ENCFF001TYS" (NES 3.06),

and

1. `` GSM1208664_batch1_chrom1_LoVo_NR2F1_PassedQC_peaks_hg19" (NES 3.05).
