# Supplementary material for: Detecting methylation signatures in neurodegenerative disease by density-based clustering of applications with reducing noise
Source: Sci Rep. 2020 Dec 17;10:22164. doi: 10.1038/s41598-020-78463-3 (PMC7747741; doi:10.1038/s41598-020-78463-3)
Supplement: Supplementary file 2 — Supplementary Information 2 [file 41598_2020_78463_MOESM2_ESM.pdf]

Detecting methylation signatures in neurodegenerative disease by DBSCAN: density-based clustering of applications with reducing noise

Saurav Mallik[1] **and Zhongming Zhao**[1,2,3,*]

[1]Center for Precision Health, School of Biomedical Informatics, The University of Texas Health Science Center at Houston, Houston, TX 77030, USA

[2]Human Genetics Center, School of Public Health, The University of Texas Health Science Center at Houston, Houston, TX 77030, USA

[3]Department of Psychiatry and Behavioral Sciences, McGovern Medical School, The University of Texas Health Science Center at Houston, Houston, TX 77030, USA

*zhongming.zhao@uth.tmc.edu

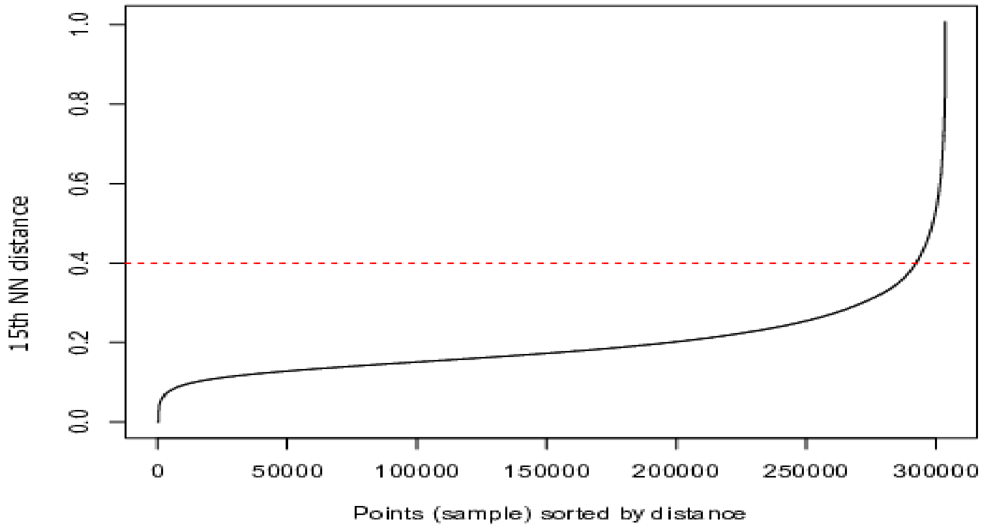

Figure S1. KNN distance plot to estimate knee point (=0.4 marked by red dotted line) used as epsilon-neighborhood (eps) in DBSCAN clustering algorithms for AD vs control (FC neurons).

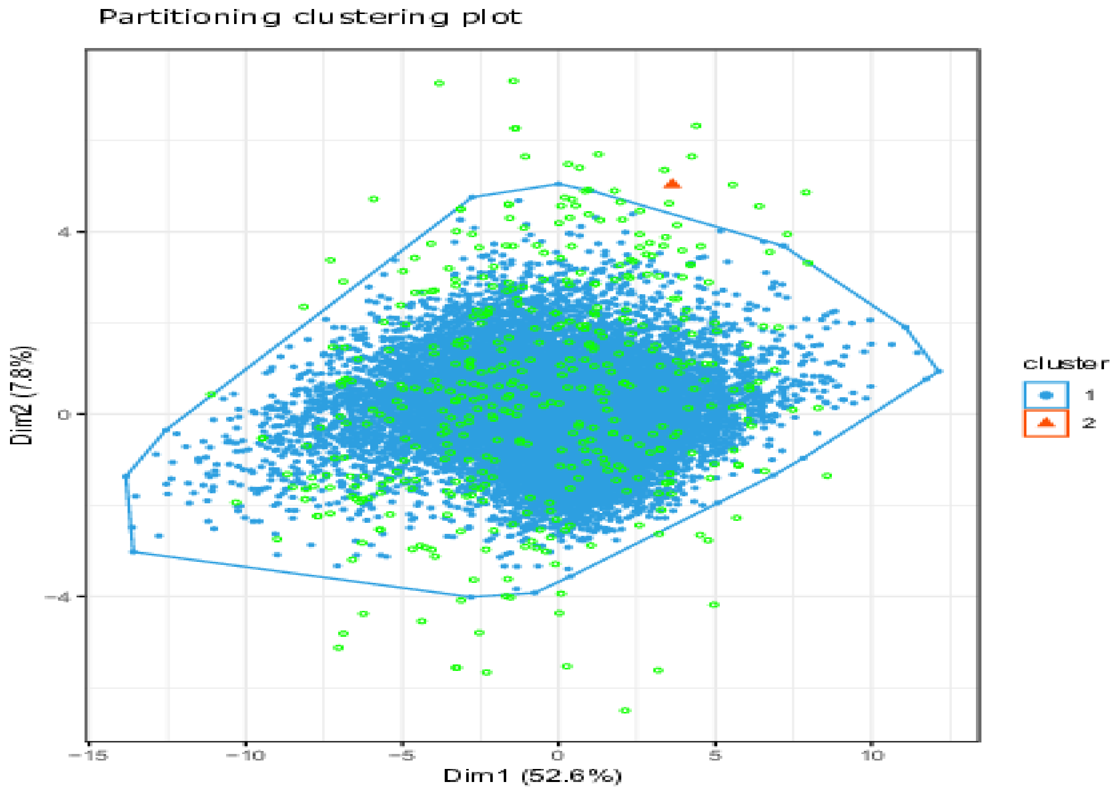

Figure S2. Partitioning clustering plot using DBSCAN clustering algorithm for AD vs control (FC neurons). Two clusters had been identified, of which the blue cluster contained 19,592 core (seed) features and 206 border features, and the orange cluster had only 10 core features. In addition, a total of 439 unclustered (outlier or noisy) features (denoted by light green dots) had been identified.

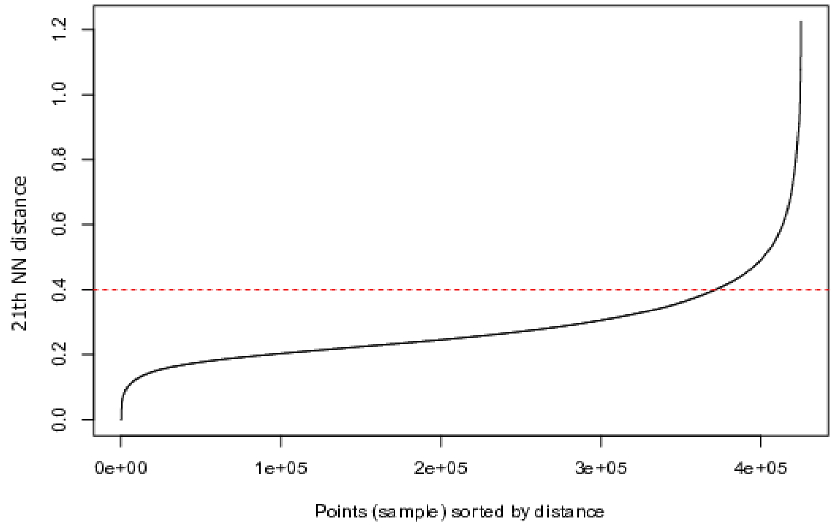

Figure S3. KNN distance plot to find knee point (=0.4 marked by red dotted line) used as EPS in DBSCAN clustering algorithms for DS vs control (FC neurons).

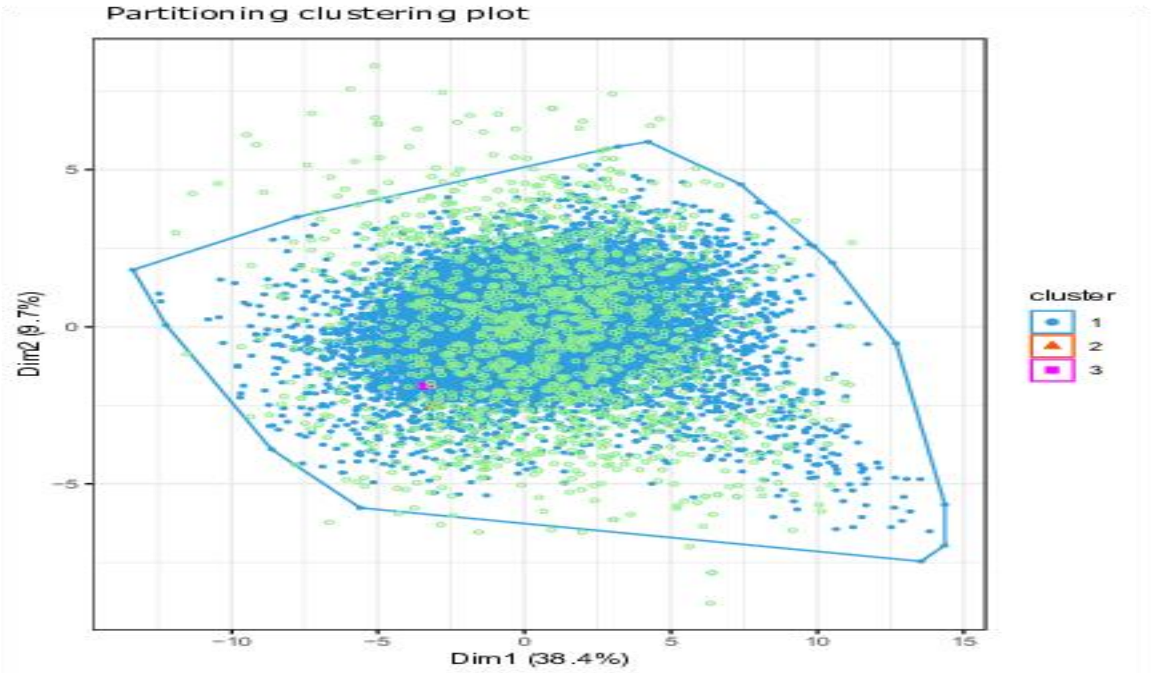

Figure S4. Partitioning clustering plot using DBSCAN clustering algorithm for DS vs control (FC neurons). Three clusters had been identified, of which the blue cluster contained 18,148 core (seed) features and 559 border features, the orange cluster had only 10 core features, and the violet cluster consisted of only 5 core features, whereas a total of 1,525 unclustered (outlier/noisy) features (denoted by light green dots) had been identified.

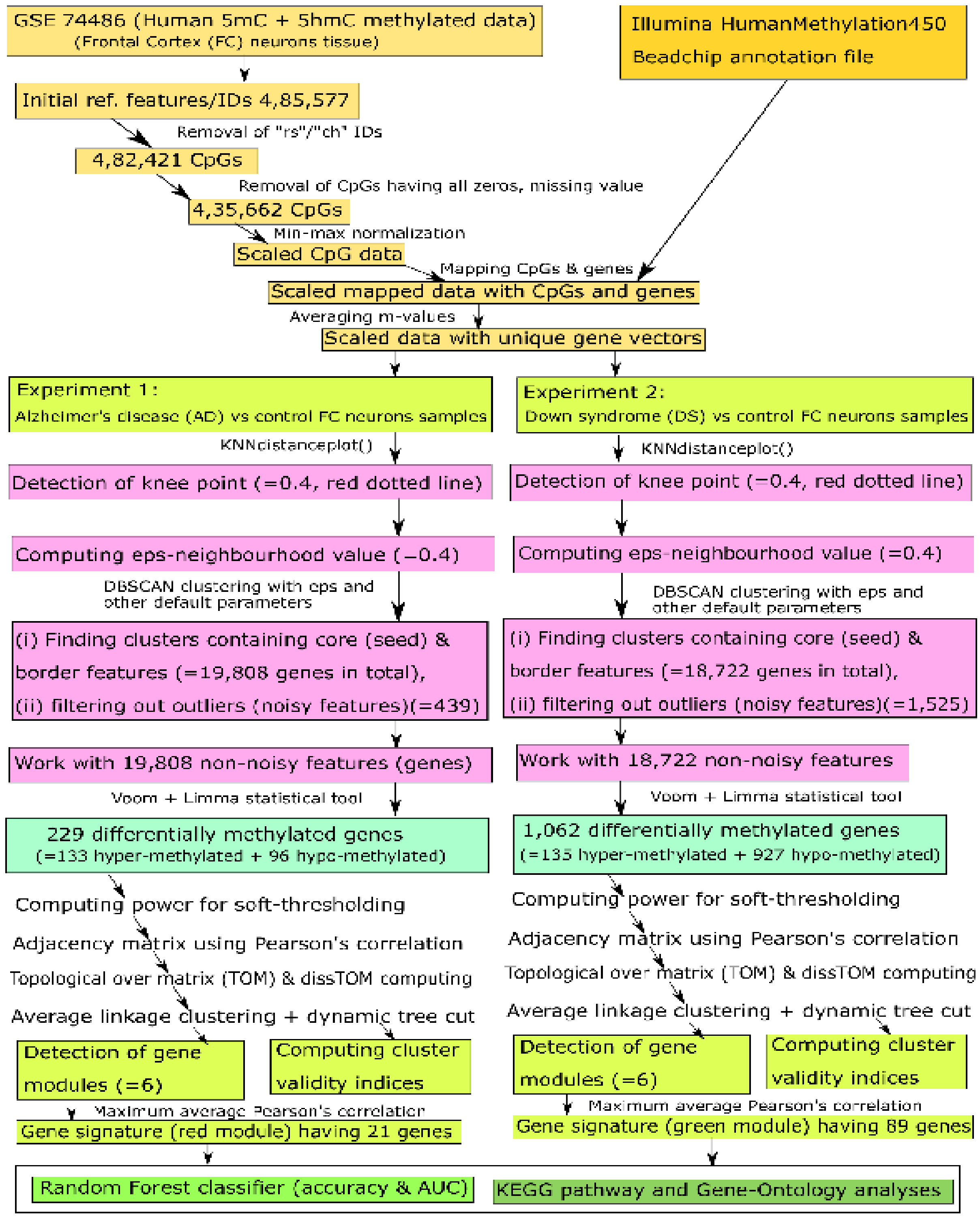

Figure S5. Detailed flowchart of the analysis.

A

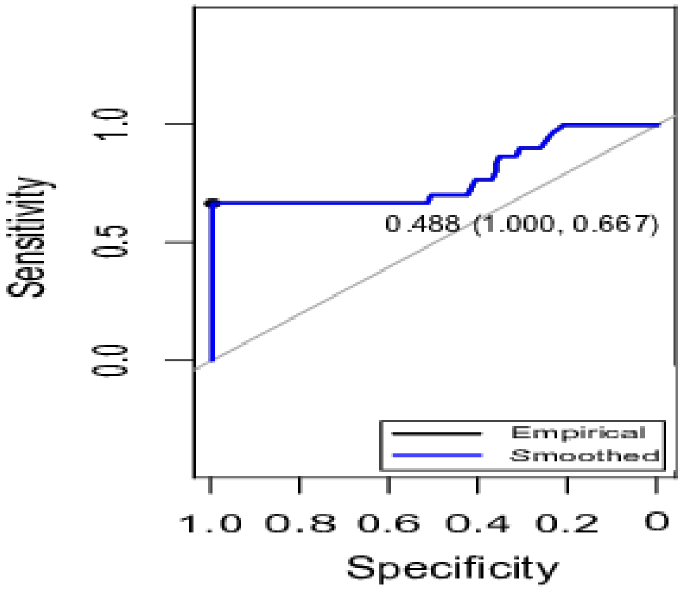

B

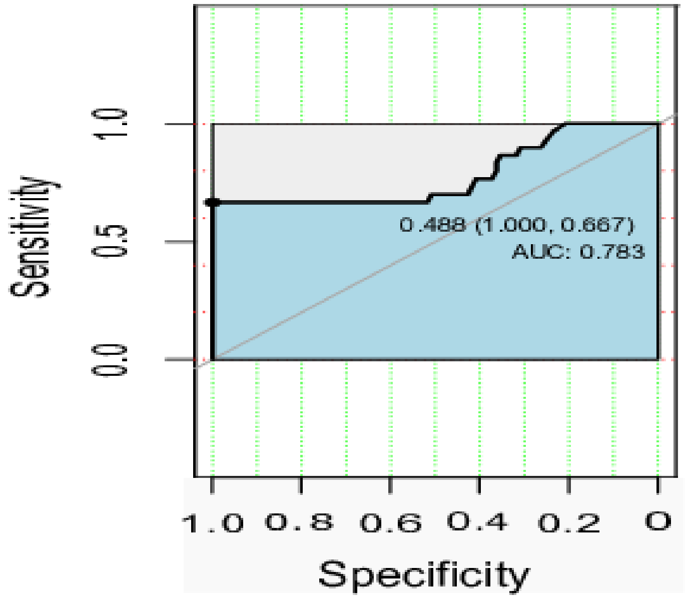

Figure S6. AUC with 4-fold cross-validation for AD vs control (FC neurons). (A) Empirical and smoothed patterns for specificity vs sensitivity plots. (B) AUC value (=0.783) computing.

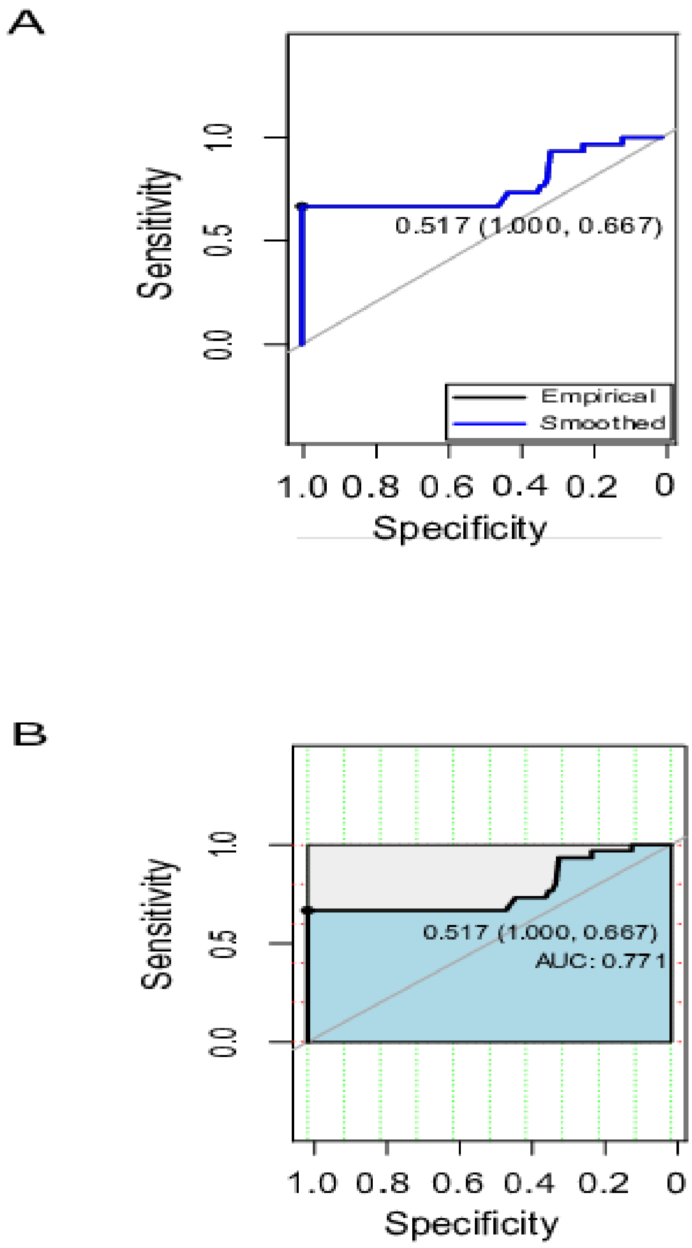

Figure S7. AUC with 5-fold cross-validation for AD vs control (FC neurons). (A) Empirical and smoothed patterns for specificity vs sensitivity plots. (B) AUC value (=0.771) computing.

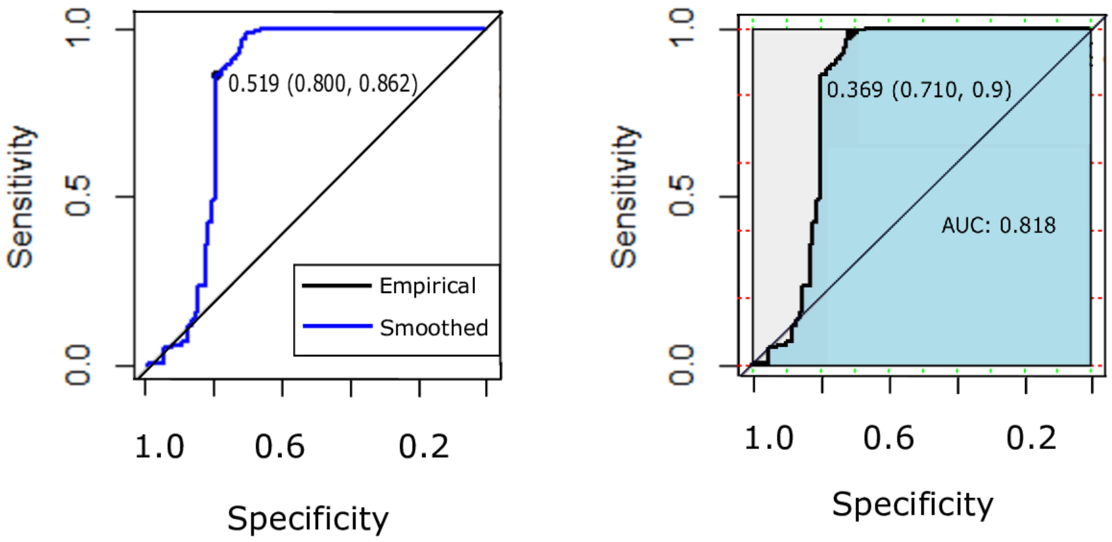

Figure S8. AUC with 8-fold cross-validation for DS vs control (Cerebellum tissue). (A) Empirical and smoothed patterns for specificity vs sensitivity plots. (B) AUC value (=0.818) computing.

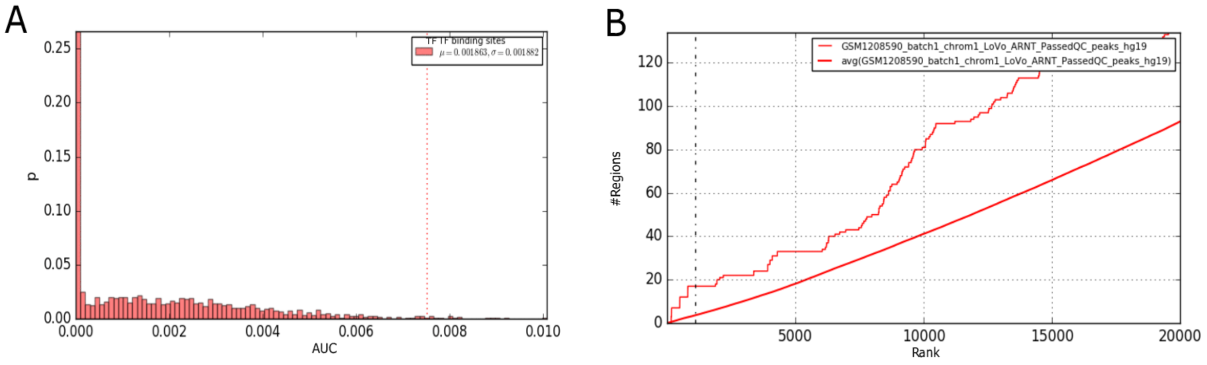

Figure S9. Plots for prediction of regulatory features and cis-regulatory modules for the 89-gene signature of DS vs control (FC Neurons). (A) Barplot of p-value vs AUC for TF binding sites. (B) Plot of #predicted regions vs rank in the feature, “GSM1208590_batch1_chrom1_LoVo_ARNT_PassedQC_peaks_hg19".

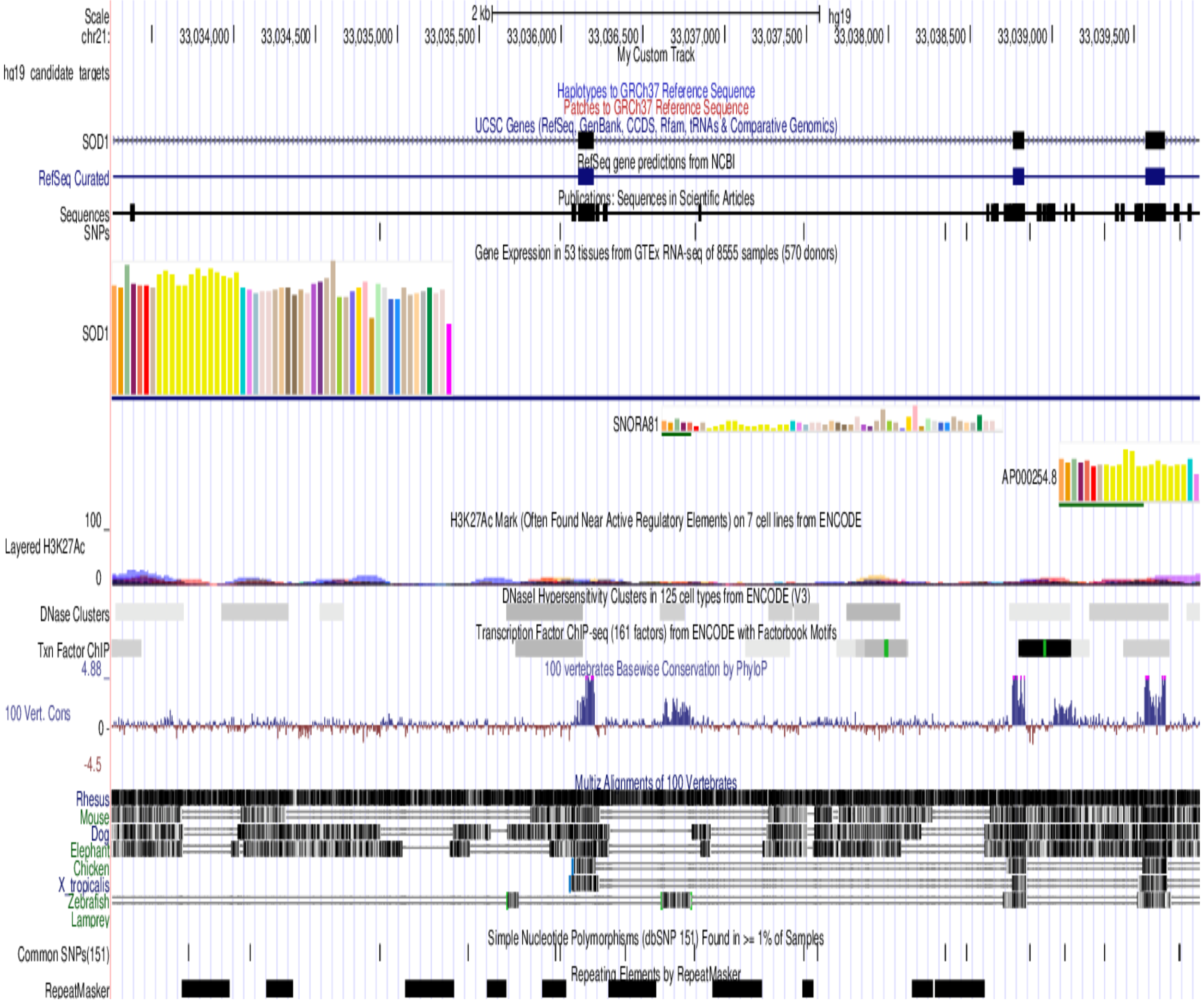

Figure S10. Significantly high ranked regions (mentioned in Figure S9) in UCSC Genome Browser for the prediction of regulatory features and cis-regulatory modules for the 89-gene signature of DS vs control (FC Neurons).

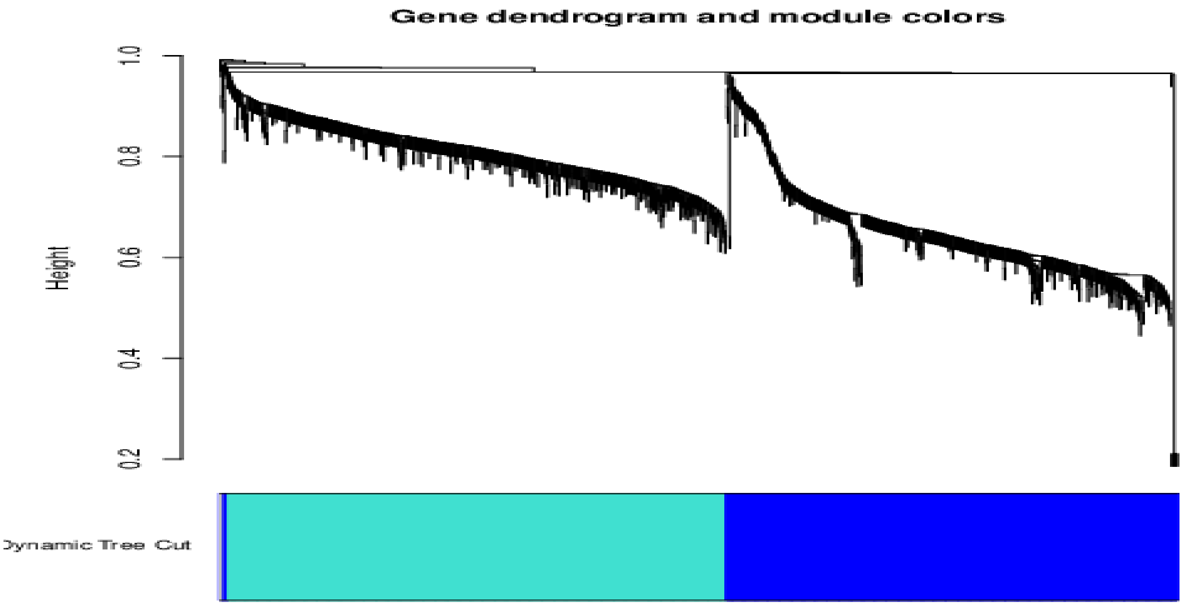

Figure S11. Dendrogram plot with color thresholding using dynamic tree cut method (32) for the comparison of DS with control (DS Cerebellum tissue). The x-axis denotes different gene modules represented by various colors, while the y-axis shows the height of the tree (dendrogram).
